# Clinical characteristics and risk factors for severe community-acquired pneumonia in hospitalized children with human metapneumovirus infection in Shanghai: a retrospective cohort study

**DOI:** 10.3389/fmed.2026.1813199

**Published:** 2026-04-30

**Authors:** Lijiao Liu, Jing Wang, Weiqin Jiang, Yuzhe Guo, Anna Cheng, Yujuan Huang

**Affiliations:** Department of Emergency, Shanghai Children’s Hospital, School of Medicine, Shanghai Jiao Tong University, Shanghai, China

**Keywords:** children, community-acquired pneumonia, human metapneumovirus, risk factors, severe pneumonia

## Abstract

**Background:**

HMPV is a leading cause of pediatric lower respiratory tract infections, second only to respiratory syncytial virus in causing severe pneumonia in children. Its clinical presentation closely mimics other common respiratory viruses, making diagnosis challenging. This study aimed to characterize the clinical profile and identify independent risk factors for severe community-acquired pneumonia (CAP) in hospitalized children with human metapneumovirus (HMPV) infection.

**Methods:**

We conducted a retrospective cohort study of 878 children hospitalized with HMPV-positive CAP at Shanghai Children’s Hospital from 2021 to 2024. Patients were classified into mild (*n* = 632) and severe (*n* = 246) groups based on established CAP criteria. Data on demographics, clinical features, laboratory findings, and concomitant pathogen detection were collected. Multivariate logistic regression was used to identify independent risk factors for severe disease.

**Results:**

The severe group comprised 28.0% of the cohort. Key clinical manifestations included fever (98.0%), cough (97.6%), wheezing (56.5%), and pulmonary crackles (84.6%). Compared to the mild group, the severe group had significantly higher rates of premature birth, wheezing, elevated inflammatory markers (NLR > 1, PCT > 1 ng/mL, CRP ≥ 50 mg/L), and co-infection with *Mycoplasma pneumoniae* (MP) (all *P* < 0.05). Multivariate analysis identified premature birth (odds ratio [OR] = 2.43, 95% CI: 1.45–4.07), wheezing (OR = 3.47, 95% CI: 2.51–4.80), NLR > 1 (OR = 1.75, 95% CI: 1.26–2.43), PCT > 1 ng/mL (OR = 2.38, 95% CI: 1.42–3.99), CRP ≥ 50 mg/L (OR = 2.20, 95% CI: 1.19–4.06), and co-infection with MP (OR = 2.17, 95% CI: 1.53–3.08) as independent risk factors for severe CAP in children with HMPV infection.

**Conclusion:**

Approximately 28% of hospitalized children with HMPV-positive CAP progress to severe disease. Premature birth, wheezing, elevated inflammatory markers, and co-infection with MP are independent risk factors, which can facilitate early risk stratification and targeted clinical management.

## Introduction

Human metapneumovirus (HMPV) is an enveloped, negative-sense single-stranded RNA virus belonging to the family Pneumoviridae. It is a globally significant pathogen responsible for acute lower respiratory tract infections (LRTIs), with particularly pronounced clinical impact in infants, older adults, and immunocompromised individuals (1). First identified in 2001 (2), HMPV has since been recognized as the second most common cause of community-acquired pneumonia (CAP) and bronchiolitis in children under 5 years of age worldwide (1). In the Pneumonia Etiology Research for Child Health (PERCH) study-a large, international, multicenter case-control study conducted across seven countries in Africa and Asia-HMPV was identified as the second leading pathogen causing severe pneumonia in children after respiratory syncytial virus (RSV) (3). Epidemiological data underscore its substantial burden: a 2018 global analysis estimated it caused approximately 1.42 million LRTI cases, 643,000 hospitalizations, and 7,700 in-hospital deaths under five (4).

Despite its substantial disease burden, management of HMPV infection remains challenging. Its symptomatology often overlaps considerably with other common respiratory viruses, such as RSV, making differential diagnosis based solely on clinical presentation difficult (5). Crucially, there are currently no licensed vaccines or specific antiviral therapies for HMPV; thus, supportive care remains the cornerstone of management (6). This therapeutic gap underscores the importance of early risk stratification tools. Although most HMPV infections follow a self-limiting course, a considerable proportion of children progress to severe CAP requiring hospitalization and, in some cases, posing life-threatening risks. While known risk factors for severe pediatric CAP exist, the precise microbial and host determinants underlying severe CAP in children with HMPV infection remain incompletely understood. Identifying these factors is critical for enabling timely intervention and optimal resource allocation in high-risk individuals.

To address this clinical gap, we conducted a retrospective cohort study analyzing clinical and epidemiological data from children (≤17 years) hospitalized with laboratory-confirmed HMPV-positive CAP at a tertiary pediatric center in Shanghai, China, between January 2021 and December 2024. We hypothesized that specific clinical and demographic parameters could serve as independent predictors of progression to severe disease. Our primary objectives were: (1) to describe the clinical and epidemiological characteristics of severe pneumonia in children with HMPV infection in our cohort; (2) to identify independent risk factors associated with progression to severe pneumonia; and (3) to propose an evidence-based framework for early clinical identification of high-risk patients to guide management strategies. Our findings provide new insights into the clinical assessment, management and disease course of severe pneumonia in children with HMPV infection, contribute to a more comprehensive understanding of this important respiratory pathogen, and offer potential directions for targeted surveillance and future interventions.

## Materials and methods

### Study design and patient cohort

This retrospective cohort study analyzed the medical records of 878 pediatric patients (aged ≤ 17 years) hospitalized at Shanghai Children’s Hospital with HMPV-positive CAP between January 2021 and December 2024. The study protocol adhered to the principles of the Declaration of Helsinki and was approved by the Institutional Review Board of Shanghai Children’s Hospital (Approval No. 2025R005). As this was a retrospective chart review involving anonymized data, the requirement for informed consent was waived.

Inclusion criteria were as follows: (i) age ≤ 17 years; (ii) a respiratory specimen (nasopharyngeal swab, sputum, or bronchoalveolar lavage fluid) testing positive for HMPV via nucleic acid amplification; and (iii) fulfillment of the diagnostic criteria for CAP as outlined in the Guidelines for the Management of CAP in Children (2024 Revised Edition) (7).

Exclusion criteria comprised: (i) a confirmed nosocomial infection; and (ii) incomplete or missing essential clinical or laboratory records.

### Clinical data acquisition and case classification

Demographic and clinical data were extracted from the hospital’s electronic medical record system. For each HMPV-positive patient, we recorded: presenting symptoms, demographic information (age, sex), comprehensive laboratory findings [including white blood cell count, hemoglobin, platelet count, C-reactive protein (CRP), liver and renal function panels, coagulation profiles, cardiac enzyme levels, procalcitonin (PCT), T-cell subset counts, and humoral immune function], documented underlying comorbidities, details of any mixed viral or bacterial infections, hospitalization parameters (length of stay, ward type), chest imaging reports, and total hospitalization costs. Based on the 2024 pediatric CAP guidelines (7), patients were stratified into two groups: severe pneumonia and non-severe (mild) pneumonia. Severe pneumonia was defined as the presence of any of the following criteria:(a) poor general condition;(b) refusal to eat or signs of dehydration;(c) altered mental status (lethargy, coma, convulsions);(d) markedly increased respiratory rate (infants > 70 breaths/min, older children > 50 breaths/min);(e) cyanosis or respiratory distress (nasal flaring, chest retractions, grunting, or central cyanosis);(f) oxygen saturation ≤ 92% on room air at rest (at sea level);(g) radiographic findings: multilobar involvement or ≥2/3 involvement of a single lobe;(h) pleural effusion;(i) extrapulmonary complications. Patients who did not meet any of these criteria were classified as having mild pneumonia.

The clinical indications of chest CT : (i) inconclusive findings on chest radiography; (ii) clinical suspicion of severe pneumonia complications, such as pulmonary consolidation, atelectasis, lung abscess, necrotizing pneumonia, or pleural effusion; and (iii) need to differentiate airway foreign bodies or structural lung disease.

### Pathogen detection methodologies

#### Respiratory viral panel

Within 24 h of admission, a respiratory specimen was collected from all patients. Nucleic acid extraction was performed using a Smart Lab Assist automated nucleic acid extraction system (Ningbo Health Gene Technologies Co., Ltd., China), and pathogen detection was conducted using a commercial multiplex polymerase chain reaction (PCR) assay with capillary electrophoresis (Ningbo Health Gene Technologies Co., Ltd., China). This panel targeted 11 viruses: HMPV, Influenza A virus (FluA, subtyped as H1N1 and H3N2), Human Adenovirus (HAdV), Human Respiratory Syncytial Virus (HRSV), Human Parainfluenza Viruses (HPIV), Human Rhinovirus (HRV), Influenza B virus (FluB), Human Coronaviruses (HCoV), and Human Bocavirus (HBoV); and two atypical bacteria: *Mycoplasma pneumoniae* (MP) and *Chlamydia pneumoniae* (CP). Multiplex reverse transcription and PCR were performed using the Thermo Fisher ProFlex™ PCR system, and amplified products were subjected to capillary electrophoresis on the ABI 3500 Dx genetic analyzer (Thermo Fisher Scientific, United States). Fragment size and peak height were analyzed using Gene Mapper (ABI Prism) software to determine pathogen detection results. For quality control, positive and negative controls provided with the kit were included in each run to monitor the entire process, including nucleic acid extraction, amplification, and capillary electrophoresis. In addition, an endogenous internal control (monitoring of internal RNA and DNA peaks) was used to assess specimen quality, ensuring the reliability of the test results. All procedures were performed strictly in accordance with the manufacturer’s instructions.

#### Serological testing for *M. pneumoniae*

Concurrently, a 2–3 mL sample of venous blood was drawn within 24 h of admission. Serum or plasma was tested for *M. pneumoniae*-specific IgM and IgG antibodies using a passive hemagglutination assay (Fujirebio Inc., Japan). An antibody titer of ≥1:160 was considered indicative of a recent or active infection.

#### Bacterial culture

Lower respiratory tract secretions were obtained via sputum induction or suction within 24–48 h of admission. Sputum samples were deemed adequate for culture if microscopic examination revealed <10 squamous epithelial cells per low-power field and >25 polymorphonuclear neutrophils per high-power field (a standard quality control measure to ensure a deep-cough specimen rather than oral contamination). Adequate specimens were cultured using both conventional agar plating and an automated microbial identification and antimicrobial susceptibility testing system (VITEK 2 Compact, bioMérieux, France). Furthermore, for patients who underwent diagnostic bronchoscopy per the Chinese Guidelines for Pediatric Flexible Bronchoscopy (2018 Edition) (8), bronchoalveolar lavage (BAL) fluid (3–5 mL) was also cultured using the same methods.

### Statistical analysis

Statistical analyses were performed using IBM SPSS Statistics software (version 27.0; IBM Corp., Armonk, NY, United States). Continuous variables are presented as median with interquartile range (IQR), categorical variables are presented as frequencies and percentages. For between-group comparisons, the non-parametric Mann-Whitney U test was used for continuous variables, while the Chi-square (χ^2^) test or Fisher’s exact test was applied for categorical variables, as appropriate. To identify independent risk factors for severe pneumonia in children with HMPV infection, variables with a *P*-value < 0.05 in univariate analyses were entered into a multivariable logistic regression model. Forward stepwise selection was applied with entry and removal thresholds set at α = 0.05 and α = 0.10, respectively. The model’s goodness-of-fit was assessed using the Hosmer-Lemeshow test. Results are reported as adjusted odds ratios (aORs) with 95% confidence intervals (CIs). A two-tailed *P*-value of < 0.05 was considered statistically significant.

## Results

### Demographic characteristics

A total of 878 pediatric patients with HMPV-positive CAP were included, comprising 429 males (48.9%) and 449 females (51.1%) (male-to-female ratio 1:1.05). The severe pneumonia group accounted for 246 cases (28.0%), with a median age of 44 months [interquartile range (IQR): 23–67 months]; within this group, 103 cases (41.9%) were male and 143 (58.1%) female, and the median hospitalization duration was 7 days (IQR: 6–9 days). The mild pneumonia group included 632 cases (72.0%), with a median age of 36 months (IQR: 24–60 months); among these, 326 cases (51.6%) were male and 306 (48.4%) female, and the median length of hospital stay was 6 days (IQR: 5–7 days) (Table 1). In the subgroup of patients with single HMPV infection (*n* = 351), the differences in clinical characteristics between mild and severe pneumonia were similar to those in the overall cohort, as detailed in Supplementary Table 1.

**TABLE 1:** Comparison of clinical characteristics between mild and severe pneumonia groups in children with HMPV^a^ infection.

Parameters^b^	Types of pneumonia		χ^2c^	*P*-value
	Mild pneumonia	Severe pneumonia		
Total (*n*, %)	632 (72)	246 (28)	–	–
Gender, males *n* (%)	326 (51.6)	103 (49.1)	2.975	0.087
Age, median (IQR), month	36 (24, 60)	44 (23, 67)	−1.545	0.122
Preterm births (*n*, %)	40 (6.3)	36 (14.6)	15.447	<0.001
Underlying diseases (*n*, %)	103 (16.3)	49 (19.9)	1.622	0.203
Wheezing (*n*, %)	180 (28.5)	139 (56.5)	60.114	<0.001
Pulmonary crackles (*n*, %)	550 (87.0)	208 (84.6)	1.009	0.315
WBC (*10∧9/L)	6.7 (4.83, 8.84)	6.24 (4.39, 9.44)	−1.195*	0.232
NE% (IQR)	33.8 (9, 54.2)	53.3 (37.9, 69.3)	−9.814*	<0.001
NLR>1 (*n*, %)	315 (49.8)	163 (66.3)	19.245	<0.001
CRP ≥ 50 mg/L (*n*, %)	31 (4.9)	31 (12.6)	15.983	<0.001
PCT>1 ng/ml (*n*, %)	41 (6.5)	44 (17.9)	26.313	<0.001
Infection type			8.807	0.003
Single HMPV infection (*n*, %)	272 (43)	79 (32.1)	–	–
Co-infections (*n*, %)	360 (57)	167 (67.9)	–	–
Comorbidities (*n*, %)				
Immune dysfunction	13 (2.1)	10 (4.1)	2.786	0.095
Coagulation dysfunction	17 (2.7)	9 (3.7)	0.578	0.447
Hypoproteinemia	15 (2.4)	12 (4.9)	3.727	0.054
Electrolyte imbalance	8 (1.3)	13 (5.3)	12.250	<0.001
Sepsis	10(1.6)	21(8.5)	25.143	<0.001
Length of hospital stay (days)	6 (5, 7)	7 (6, 9)	−7.376*	<0.001

*^a^*HMPV, human metapneumovirus.

*^b^*WBC, white blood cell count; NE%, neutrophil percentage; NLR, neutrophil-to-lymphocyte ratio; CRP, C-reactive protein; PCT, procalcitonin.

^c^* represents the Z-value.

### Seasonal and interannual variation

Severe HMPV-positive CAP exhibited distinct seasonal clustering patterns, except for an unusual 2023 epidemic, with winter as the primary peak season in other years: 2021 had 25 cases (23 [92.0%] in winter); 2022 had 62 cases (47 [75.8%] in winter); 2023 had 70 cases distributed across seasons (15 [21.4%] spring, 29 [41.4%] summer, 18 [25.7%] autumn, 8 [11.4%] winter); and 2024 had 89 cases (58 [65.2%] in winter) (Supplementary Table 4).

### Underlying medical conditions

Among the 246 severe cases, 49 (19.9%) had underlying conditions, including congenital heart disease (29 [11.8%]), epilepsy (10 [4.1%]), cerebral palsy (7 [2.8%]), brain hypoplasia (7 [2.8%]), developmental delay (7 [2.8%]), congenital airway developmental anomalies (5 [2.0%], 3 bronchopulmonary dysplasia and 2 congenital laryngeal cartilage softening), chromosomal abnormalities (4 [1.6%], including 1 Down syndrome), coronary artery anomalies (4 [1.6%]), severe malnutrition (4 [1.6%]), bronchial asthma (3 [1.2%]), acute lymphoblastic leukemia (2 [0.8%]), liver transplant status (2 [0.8%]), scoliosis (2 [0.8%]), and single instances (each 0.4%) of nephrotic syndrome, Wilms tumor, pulmonary hypertension, aortic stenosis, pseudohypoaldosteronism, methylmalonic acidemia, cerebral tendon cholesterol deposits, and multiple intestinal polyps.

### Clinical features, complications, and laboratory/imaging findings

Clinically, 241 severe cases (98.0%) presented with fever, including 170 (69.1%) with high fever (39.1°C–41°C); cough occurred in 240 cases (97.6%), pulmonary crackles in 208 (84.6%), wheezing in 139 (56.5%), vomiting in 66 (26.8%), diarrhea in 58 (23.6%), and seizures in 12 (4.9%). Concurrent complications included acute respiratory failure (101 [41.1%]), neutropenia (25 [10.2%]), sepsis (21 [8.5%]), electrolyte imbalance and liver injury (13 [5.3%]), hypoalbuminemia (12 [4.9%]), immune dysfunction (10 [4.1%]), coagulation abnormalities (9 [3.7%]), myocardial injury (8 [3.3%]), shock (7 [2.8%]), gastrointestinal dysfunction and agranulocytosis (5 [2.0%]), and renal insufficiency (3 [1.2%]).

Laboratory findings showed a median WBC count of 6.24 × 10^9^/L (IQR: 4.39–9.44 × 10^9^/L), with 34 cases (13.8%) having WBC > 12 × 10^9^/L; median neutrophil percentage was 53.3% (IQR: 37.9–69.3%). Elevated CRP was observed in 157 cases (63.8%), including 78 (31.7%) with CRP ≥ 20 mg/L and 31 (12.6%) with CRP ≥ 50 mg/L; 138 cases (54.1%) had elevated erythrocyte sedimentation rate (ESR), 68 (27.6%) had PCT > 0.5 ng/mL, and 44 (17.9%) had PCT > 1 ng/mL. Imaging revealed chest X-rays in 11 cases (4.5%) and chest CT in 235 (95.5%); multilobar inflammation was seen in 230 cases (93.5%), unilobar in 16 (6.5%), pulmonary consolidation in 89 (36.2%), pleural thickening in 33 (13.6%), pleural effusion in 26 (10.6%), bilateral atelectasis in 13 (5.3%), and bronchiectasis in 1 (0.4%).

### Mixed infections

Among the 246 children with severe HMPV-positive pneumonia, single HMPV infection was identified in 79 cases (32.1%), whereas mixed infections involving other pathogens were detected in 167 cases (67.9%). Of these mixed infections, 89 cases (53.3%) involved co-infection with one additional pathogen, 56 cases (33.5%) with two pathogens, 18 cases (10.8%) with three pathogens, 3 cases (1.8%) with four pathogens, and 1 case (0.6%) with five pathogens. A total of 169 cases had co-infection with atypical bacteria and viruses, including: MP in 93 cases (55.7%), FluB in 17 cases (10.2%), HRV in 17 cases (10.2%), HRSV in 13 cases (7.8%), HAdV in 9 cases (5.4%), HPIV in 8 cases (4.8%), HBoV in 7 cases (4.2%), HCoV in 2 cases (1.2%), CP species in 2 cases (1.2%), and FluA in 1 case (0.6%). In addition, bacterial co-infections were detected in 108 cases, including: *Haemophilus influenzae (H. influenzae)* in 47 cases (28.1%), *Streptococcus pneumoniae* in 15 cases (9.0%), *Staphylococcus aureus* in 13 cases (7.8%), *Moraxella catarrhalis* in 7 cases (4.2%), *Pseudomonas aeruginosa* in 6 cases (3.6%), *Escherichia coli* in 5 cases (3.0%), *Klebsiella pneumoniae* in 4 cases (2.4%), and other bacterial species in 11 cases (6.6%). One case (0.6%) had a fungal co-infection with *Candida parapsilosis*. The comparison of co-infection pathogens between the severe and mild pneumonia groups is shown in Supplementary Table 2.

### Treatment and outcomes

Among the 246 pediatric patients with severe pneumonia in the context with HMPV infection, 93 (37.8%) required non-invasive ventilation for respiratory support, including nasal cannula, face mask, high-flow nasal oxygen therapy, and bilevel positive airway pressure (BiPAP), seventy-two patients (29.3%) were admitted to the intensive care unit (ICU), and 34 cases (13.8%) required invasive mechanical ventilation. Antibiotic therapy was administered to 245 patients (99.6%). Additional treatments included intravenous systemic glucocorticoids in 135 cases (54.9%), human immunoglobulin infusion in 25 cases (10.2%), magnesium sulfate for bronchodilation and spasm relief in 27 cases (11.0%), fiberoptic bronchoscopy in 47 cases (19.1%), and continuous renal replacement therapy (CRRT) in 3 cases (1.2%). The median length of hospital stay was 7 days [interquartile range (IQR): 6–9 days]. Clinical outcomes were as follows: 238 patients (96.7%) recovered and were discharged, three patients (1.2%) died, and five patients (2.0%) left the hospital voluntarily due to personal or social reasons, including one patient who was discharged while still receiving tracheal intubation and one patient who requested extubation prior to discharge.

### Analysis of risk factors for severe pneumonia in children with HMPV infection

Compared with the mild pneumonia group, the severe pneumonia group had significantly higher proportions of preterm birth, wheezing, mixed infections, and concomitant electrolyte disturbances or sepsis (all *P* < 0.05). However, no statistically significant differences were observed between the two groups in age, pulmonary crackles, underlying conditions, immune dysfunction, or coagulation abnormalities (*P* = 0.203) (Table 1). Laboratory findings showed that a neutrophil-to-lymphocyte ratio (NLR) > 1, PCT > 1 ng/mL, and CRP ≥ 50 mg/L were significantly more prevalent in the severe pneumonia group than in the mild pneumonia group (all *P* < 0.001) (Table 1). Regarding mixed pathogens, the severe pneumonia group had a higher proportion of concurrent *H. influenzae* and MP infections than the mild pneumonia group (all *P * < 0.05) (Table 2). With respect to treatment, the severe pneumonia group had higher rates of systemic glucocorticoid use, human immunoglobulin therapy, and fiberoptic bronchoscopy; moreover, the duration of hospitalization was longer in the severe pneumonia group, with all differences reaching statistical significance (all *P* < 0.05) (Table 3). Furthermore, within the severe pneumonia group, the proportion of patients undergoing fiberoptic bronchoscopy was significantly higher in the co-infection group than in the single HMPV infection group (Supplementary Table 3).

**TABLE 2:** Comparison of co-infections between mild and severe pneumonia groups in children with HMPV^a^ infection.

Pathogen^b^	Types of pneumonia		χ^2^	*P*-value
	Mild pneumonia	Severe pneumonia		
Total (*n*, %)	632 (72)	246 (28)	–	–
Atypical bacteria and viruses				
*MP* (*n*, %)	151 (23.9)	98 (39.8)	17.08	<0.001
FluB (*n*, %)	75 (11.9)	17 (6.9)	3.15	0.071
HRV (*n*, %)	68 (10.8)	17 (6.9)	3.00	0.083
HRSV (*n*, %)	27 (4.3)	13 (5.3)	0.417	0.518
HAdV (*n*, %)	20 (3.2)	9 (3.7)	0.135	0.713
HPIV (*n*, %)	18 (2.8)	8 (3.3)	0.101	0.751
HBoV (*n*, %)	13 (2.1)	7 (2.8)	0.495	0.482
Bacterial pathogens (*n*, %)				
*H. influenzae*	83 (13.1)	47 (19.1)	5.008	0.025
*Streptococcus pneumoniae*	51 (8.1)	15 (6.1)	0.991	0.320
*Staphylococcus aureus*	23 (3.6)	13 (5.3)	1.219	0.270
*Klebsiella pneumoniae*	32 (5.1)	7 (2.8)	2.052	0.152

*^a^*HMPV, human metapneumovirus.

*^b^MP*, *Mycoplasma pneumoniae*; HRV, Human Rhinovirus; HAdV, Human Adenovirus; HRSV, Human Respiratory Syncytial Virus; HPIV, Human Parainfluenza Virus; FluB, Influenza B virus; HBoV, Human Bocavirus; *H. influenzae*, *Haemophilus influenzae*.

**TABLE 3:** Comparison of treatment outcomes between mild and severe pneumonia groups in children with HMPV^a^ infection.

Group	*n*	Glucocorticoids (*n*, %)	Human immunoglobulin (*n*, %)	Fiber optic bronchoscopy treatment (*n*, %)	Antibiotics (*n*, %)	Length of hospital stay (IQR)
Mild	632	145 (22.9)	36 (5.7)	20 (3.2)	624 (98.7)	6 (5, 7)
Severe	246	135 (54.9)	25 (10.2)	47 (19.1)	245 (99.6)	7 (6, 9)
χ^2b^		83.142	5.464	57.806	0.670	−7.376*
*P*		<0.001	0.019	<0.001	0.413	<0.001

*^a^*HMPV, human metapneumovirus.

^b^* represents the Z-value.

Variables with *P* < 0.05 in univariate analysis (preterm birth, wheezing, NLR > 1, CRP ≥ 50 mg/L, PCT > 1 ng/mL, co-infection, electrolyte disturbance, and sepsis), common co-pathogens with statistical significance in univariate analysis (MP, *H. influenzae*), and potential confounders (age, sex, and underlying diseases) were included in the multivariable binary logistic regression analysis. The following factors were identified as independently associated with severe pneumonia in children with HMPV infection: preterm birth (odds ratio [OR] = 2.43, 95% confidence interval [CI] 1.45–4.07, *P* = 0.001), wheezing (OR = 3.47, 95% CI 2.51–4.80, *P * < 0.001), NLR > 1 (OR = 1.75, 95% CI 1.26–2.43, *P* = 0.001), PCT > 1 ng/mL (OR = 2.38, 95% CI 1.42–3.99, *P* = 0.001), CRP ≥ 50 mg/L (OR = 2.20, 95% CI 1.19–4.06, *P* = 0.012), and mixed *MP* infection (OR = 2.17, 95% CI 1.53–3.08, *P* < 0.001). Other candidate variables were not retained in the final model (*P* > 0.05) (Table 4).

**TABLE 4:** Multivariate binary logistic regression analysis of risk factors for severe pneumonia in children with HMPV^a^ infection.

Variables^b^	β-value	Standard error	Wald-value	*P*-value	OR (95% CI)^c^
Premature birth	0.89	0.27	11.20	0.001	2.43 (1.45∼4.07)
Wheezing	1.24	0.17	56.18	<0.001	3.47(2.51∼4.80)
NLR > 1	0.56	0.17	11.46	0.001	1.75 (1.26∼2.43)
CRP ≥ 50 mg/L	0.79	0.31	6.38	0.012	2.20 (1.19∼4.06)
PCT > 1 ng/ml	0.87	0.27	10.70	0.001	2.38 (1.42∼3.99)
Co-infection with *MP*	0.78	0.18	19.21	<0.001	2.17 (1.53∼3.08)

*^a^*HMPV, human metapneumovirus.

*^b^*NLR, neutrophil-to-lymphocyte ratio; CRP, C-reactive protein; PCT, procalcitonin; *MP*, *Mycoplasma pneumoniae*.

*^c^*OR, odds ratio; CI, confidence interval.

## Discussion

HMPV is a significant causative pathogen of CAP. The clinical features, risk factors, and prognostic management of severe pneumonia in children with HMPV infection remain key areas of investigation in pediatric infectious diseases. Based on clinical data from 878 pediatric patients with HMPV-positive CAP, the present study systematically analyzed epidemiological characteristics, underlying disease profiles, clinical manifestations, laboratory findings, mixed infection patterns, and treatment outcomes in the severe pneumonia cohort. Multivariate logistic regression identified independent risk factors for progression to severe disease, thereby offering evidence-based insights to support early recognition and targeted intervention.

With respect to seasonal trends, severe pneumonia in children with HMPV infection generally exhibits a “winter peak” pattern, except for the atypical epidemic profile observed in 2023. During that year, disease incidence remained high throughout the calendar year, reaching its maximum in the summer months; in contrast, winter consistently represented the principal peak period in other years. This overall seasonal distribution is consistent with the “winter–spring epidemic” pattern reported in most previous studies (9, 10). The anomalous summer peak in 2023 is hypothesized to be linked to the implementation and subsequent relaxation of non-pharmaceutical interventions (NPIs) during the COVID-19 pandemic in 2022. Earlier investigations have demonstrated that both the introduction and withdrawal of NPIs can alter the epidemiological dynamics of multiple respiratory viruses, including HMPV (11–13). International researchers first documented an off-seasonal HMPV outbreak in June–July 2021 following the lifting of NPIs, with the epidemic peak shifting into summer (14, 15). Studies conducted in China have likewise reported a blunted seasonal pattern for HMPV in 2023 after the removal of interventions (12, 16). NPIs may diminish population exposure to pathogens and delay the accumulation of natural immunity-effects that can culminate in an “immunity debt” (17). This distinctive finding underscores that environmental factors, such as shifts in epidemic control policies, and variations in population immunity substantially influence the epidemiological cycles of HMPV infection.

In the present study, the proportion of hospitalized children with HMPV-positive CAP who progressed to severe pneumonia was 28.0% (246/878). This proportion is consistent with previous reports from China and internationally. Studies from China have reported severe proportions ranging from 21.4% to 38.7%, including 21.4% (154/721) in Wenzhou (18), 38.7% (46/119) in Hebei (19), and 35.5% (102/287) in Hangzhou (20). A study from Turkey reported a severe proportion of 21.1% (20/95) among children with HMPV-positive pneumonia (21). These variations likely reflect differences in study populations, geographic regions, diagnostic criteria, and co-infection rates. Nevertheless, the 28.0% severe proportion in this study falls within the reported range, supporting the representativeness of our cohort.

In this study, 49 children (19.9%) with severe pneumonia had identifiable underlying conditions, the most prevalent being congenital heart disease (11.8%), neurodevelopmental disorders(epilepsy [4.1%] and cerebral palsy/developmental delay [2.8%]), airway developmental abnormalities (2.0%), malnutrition (1.6%), and chromosomal abnormalities (1.6%). This profile is highly consistent with previous reports, confirming that underlying comorbidities represent significant risk factors for severe disease in children with HMPV infection (22–24). Children with congenital heart disease are predisposed to respiratory failure due to pulmonary congestion and compromised oxygen delivery (25, 26). Those with neurological impairments frequently exhibit dysphagia and diminished cough reflex, resulting in secretion retention and recurrent microaspiration (27). Anatomical airway anomalies directly impair ventilation through airway narrowing, whereas malnutrition or immunodeficiencies associated with chromosomal abnormalities markedly attenuate host defense against infection. Consequently, in children with HMPV infection who have these comorbidities, clinicians should maintain heightened vigilance and initiate early intervention to mitigate progression to severe disease.

The clinical presentation in the severe pneumonia cohort was dominated by typical pneumonic features, notably fever (98.0%, with 69.1% exhibiting high fever of 39.1 °C–41 °C), wheezing (56.5%), and pulmonary crackles (84.6%). Non-specific systemic manifestations-including vomiting (26.8%), diarrhea (23.6%), and seizures (4.9%)-were also frequent, highlighting the importance of comprehensive evaluation of systemic inflammatory responses in critically ill pediatric patients. Complication rates were markedly elevated in severe cases: acute respiratory failure (41.1%), sepsis (8.5%), electrolyte disturbances and hepatic injury (5.3%), and myocardial injury (3.3%). These complications serve as direct indicators of disease severity. Respiratory failure arises from a core mechanism of ventilation-perfusion mismatch, where an imbalance between airflow to alveoli and blood flow through pulmonary capillaries impairs efficient gas exchange, leading to hypoxemia and/or hypercapnia (28). Meanwhile, sepsis and myocardial injury suggest systemic inflammatory response syndrome, which may be associated with dysregulated immune responses in children with HMPV infection (29, 30). Laboratory analyses revealed several abnormalities: neutropenia (10.2%), hypoalbuminemia (4.9%), and immune dysfunction (4.1%). These findings corroborate that critically ill children experience immune dysregulation alongside deteriorating nutritional status. Such pathophysiological disturbances may interact to establish a deleterious cycle of “infection - immunosuppression-secondary injury.”

In this study, mixed infections were detected in 67.9% of children with severe pneumonia in the context of HMPV infection; among these, 53.3% had co-infection with one additional pathogen and 33.5% with two pathogens. These findings suggest that multipathogen synergy plays a pivotal role in the pathogenesis of severe pneumonia in children with HMPV infection. Previous studies have demonstrated that HMPV infection increases susceptibility to secondary bacterial reinfection, thereby elevating morbidity and mortality (29). This may result from opportunistic bacterial colonization and toxin release within an immunocompromised microenvironment. Furthermore, following co-infection with MP, alterations in host immune responses may further impair pathogen clearance by interacting with HMPV-induced “immune paralysis” (31, 32). The convergence of these mechanisms can disrupt immune homeostasis and amplify inflammatory cascades. Consistent with this proposed pathway, multivariate analysis identified “co-infection with MP” as an independent risk factor for severe pneumonia in children with HMPV infection (*P* < 0.001), whereas overall co-infection was not retained in the final model. Notably, MP was the most common co-pathogen in the severe group, accounting for 58.7% of co-infections. These findings suggest that the impact of co-infection on disease severity in this cohort may be largely attributable to MP. Accordingly, in pediatric patients with suspected HMPV pneumonia-particularly those with severe disease-comprehensive pathogen detection should be actively pursued to guide pathogen-directed antimicrobial therapy.

Among the cohort, 93 children (37.8%) received non-invasive ventilation, while 34 (13.8%) required invasive mechanical ventilation. These figures indicate that approximately half of severely affected children exhibited moderate-to-severe ventilatory dysfunction, aligning with the high prevalence of respiratory failure complications. Respiratory support remains the cornerstone of therapy for severe pneumonia in children with HMPV infection: early non-invasive ventilation reduces the need for endotracheal intubation, whereas mechanical ventilation is reserved for refractory hypoxemia or respiratory muscle fatigue. Immunomodulatory agents were also employed (corticosteroids in 54.9% and intravenous immunoglobulin in 10.2%) highlighting a clinical emphasis on mitigating hyperinflammation. When comparing treatment modalities between patients with single HMPV infection and those with co-infection within the severe pneumonia group, patients with co-infection were significantly more likely to undergo fiberoptic bronchoscopy, whereas no significant differences were observed in the use of corticosteroids, intravenous immunoglobulin, antibiotics, or length of hospital stay. Clinically, this suggests that among children who develop severe pneumonia, the presence of co-infection may be associated with a greater need for invasive diagnostic procedures, reflecting the increased clinical complexity in this subgroup.

Multivariate logistic regression identified six independent predictors of severe pneumonia in children with HMPV infection: preterm birth, wheezing, NLR > 1, PCT > 1 ng/mL, CRP ≥ 50 mg/L, and co-infection with MP. Preterm birth emerged as a notable risk factor despite the study population consisting of preschool-aged children (median age 44 months, range 23–67). This suggests that the long-term respiratory sequelae of prematurity, such as bronchopulmonary dysplasia or persistent immune dysregulation, continue to confer heightened vulnerability during the preschool years, a critical window for airway and immune system maturation. Wheezing denotes airway hyperresponsiveness, which predisposes to exacerbated ventilation impairment through bronchospasm and mucus plugging following infection (33). Elevated inflammatory markers (NLR > 1, PCT > 1 ng/mL, CRP ≥ 50 mg/L, all *P* < 0.05) reflect distinct aspects of systemic inflammation: NLR signifies an imbalance between neutrophilic inflammation and lymphocytic regulation; PCT is indicative of bacterial infection or severe inflammation; CRP represents nonspecific acute-phase reactivity. Their concurrent elevation denotes a robust systemic inflammatory response tightly linked to disease severity. Moreover, synergistic MP-HMPV co-infection intensifies the inflammatory cascade, resulting in greater tissue injury. These risk factors are readily obtainable clinical parameters that can inform predictive models for early stratification and management. For children with HMPV infection and ≥1 risk factor, close monitoring of vital signs, inflammatory indices, and oxygenation is advised, with prompt initiation of respiratory support or escalation of antimicrobial therapy as warranted.

This study has several limitations. First, its single-center retrospective design may introduce selection bias. Second, HMPV subtyping was not performed, precluding in-depth analysis of viral molecular epidemiology. Third, the diagnosis of HMPV infection was based on PCR detection in respiratory specimens. However, detection of viral nucleic acid in respiratory specimens does not necessarily imply etiological causation, particularly given the high co-infection rate (67.9% in this study). Moreover, some patients had both viral and bacterial co-infections, making the groups not mutually exclusive, and further stratification would have resulted in insufficient sample sizes. Therefore, we did not further stratify co-infections into viral and bacterial subgroups. Although we adjusted for co-infection in the multivariable analysis and performed subgroup analyses in patients with single HMPV infection, the potential confounding effect of co-infection on the interpretation of the results cannot be completely excluded. In addition, bacterial co-infection may significantly influence inflammatory markers such as NLR, CRP, and PCT, further complicating the interpretation of the findings. Accordingly, the results should be interpreted with caution. Fourth, *Mycoplasma pneumoniae* co-infection was diagnosed using serological criteria (antibody titer ≥ 1:160) and/or pharyngeal swab PCR. Serological methods cannot reliably distinguish between active infection and prior exposure; antibody titers in children may persist for several months or longer following infection, potentially leading to overestimation of active infection. PCR detects the presence of pathogen nucleic acid but does not differentiate between viable and non-viable organisms, and may also detect asymptomatic carriage. Although the combined use of both methods was intended to improve diagnostic sensitivity, the potential for misclassification bias cannot be completely excluded. Future studies using paired acute and convalescent serum antibody titers (e.g., a four-fold rise) in conjunction with more sensitive molecular diagnostic techniques are warranted to more accurately identify active infection. Fifth, in this study, bacterial co-infections were identified using semi-quantitative culture criteria (colony count ≥ +++) and the 48-h cutoff after admission to distinguish colonization from infection and community-acquired from hospital-acquired infections as much as possible. However, the semi-quantitative threshold cannot completely exclude the possibility of colonization for certain bacteria (e.g., *H. influenzae*, *S. pneumoniae*) that commonly reside in the upper respiratory tract of children. The 48-h cutoff cannot completely exclude infections with longer incubation periods or colonization present before admission that manifests after admission. Therefore, the results should be interpreted with caution. Sixth, the thresholds for inflammatory markers (NLR > 1, PCT > 1 ng/mL, CRP ≥ 50 mg/L) used in this study were based on previous literature and were not validated by ROC analysis in our cohort. Although these thresholds are widely used in pediatric pneumonia research, they may not be optimal for our study population. Future studies with larger sample sizes are warranted to determine the optimal cut-offs for these markers using ROC analysis. Seventh, the diagnosis of “immune dysfunction” in this study was based on the comprehensive clinical judgment of attending physicians recorded in the discharge diagnosis, rather than a unified immunological marker threshold set by the investigators. Although all patients underwent standardized cellular immunity (lymphocyte subset analysis by flow cytometry) and humoral immunity (immunoglobulin quantification) testing, diagnostic criteria for immune dysfunction may have varied among physicians, and mild immune abnormalities may not have been documented. Therefore, the reported prevalence of immune dysfunction may be subject to bias. Future prospective studies should adopt standardized immunological assessment protocols (e.g., uniform measurement of immunoglobulins and lymphocyte subsets with predefined diagnostic thresholds) to validate our findings. Future large-scale, multicenter, prospective studies incorporating molecular diagnostic techniques should prioritize addressing these limitations.

## Conclusion

A considerable proportion of children with HMPV-positive community-acquired pneumonia progress to severe disease, closely linked to underlying comorbidities, mixed infections, dysregulated inflammation, and host susceptibility factors such as preterm birth and wheezing. Clinicians should monitor seasonal epidemiological patterns closely, identify high-risk patients early, and apply targeted interventions to improve outcomes.

## Data Availability

The raw data supporting the conclusions of this article will be made available by the authors, without undue reservation.
