# Supplementary material for: Clinical characteristics and risk factors for severe community-acquired pneumonia in hospitalized children with human metapneumovirus infection in Shanghai: a retrospective cohort study
Source: Front Med (Lausanne). 2026 Apr 30;13:1813199. doi: 10.3389/fmed.2026.1813199 (PMC13171476; doi:10.3389/fmed.2026.1813199)
Supplement: Supplementary file 1 [file Supplementary_file_1.docx]

**Supplementary Material：**

Supplementary Table S1 shows the comparison of clinical characteristics between mild and severe pneumonia groups in children with single HMPV infection (n=351). This subgroup analysis was performed to evaluate the independent contribution of HMPV to disease severity by excluding cases with co-infection. Preterm birth, wheezing, NLR>1, CRP≥50 mg/L, and PCT>1 ng/mL remained significantly associated with severe pneumonia, supporting the independent role of HMPV.

Supplementary Table S2 presents the distribution of co-infecting pathogens in children with HMPV co-infection, stratified by disease severity. The denominator for each group is the number of children with co-infection (mild group, n=360; severe group, n=167). MP was the most common co-pathogen, particularly in the severe group (58.7% vs. 41.9%, *P*<0.001).

**TABLE 1**. Comparison of clinical characteristics between mild and severe pneumonia groups in children with single HMPV infection^a^

| Parameters^b^ | Types of Pneumonia | | | χ^2c^ | *P-value* |
| --- | --- | --- | --- | --- | --- |
|  | Mild pneumonia（n=272） | Severe pneumonia（n=79） |  | |  |
| Gender, males n (%) | 144（52.9） | 31（39.2） | | 3.04 | 0.081 |
| Age, median (IQR), month | 36（24，60） | 45（24，55） | | -0.19* | 0.851 |
| Preterm births（n，%） | 18（6.6） | 10（12.7） | | 4.60 | 0.041 |
| Wheezing（n，%） | 79（29.0） | 50（63.3） | | 30.89 | ＜0.001 |
| NLR＞1 | 133（48.9） | 49（62.0） | | 4.23 | 0.042 |
| CRP≥50mg/L | 7（2.6） | （8.9） | | 6.32 | 0.02 |
| PCT＞1ng/ml | 18（6.8） | 14（18.9） | | 9.87 | 0.003 |

^a^HMPV: Human metapneumovirus. ^b^NLR: Neutrophil-to-lymphocyte ratio; CRP: C-reactive protein; PCT: Procalcitonin.^c^* represents the Z-value

**TABLE 2**. Comparison of pathogen distribution between mild and severe pneumonia groups in children with HMPV co-infection^a^

| Pathogen^b^ | Types of Pneumonia | | χ^2^ | *P-value* |
| --- | --- | --- | --- | --- |
|  | Mild pneumonia（n=360） | Severe pneumonia（n=167） |  |  |
| Atypical bacteria and viruses |  |  |  |  |
| MP（n，%） | 151（41.9） | 98（58.7） | 12.98 | ＜0.001 |
| FluB（n，%） | 75（20.8） | 17（10.2） | 8.72 | 0.003 |
| HRV（n，%） | 68（18.9） | 17（10.2） | 6.35 | 0.012 |
| HRSV（n，%） | 27（7.5） | 13（7.8） | 0.01 | 0.910 |
| HAdV（n，%） | 20（5.6） | 9（5.4） | 0.01 | 0.713 |
| HPIV（n，%） | 18（5.0） | 8（4.8） | 0.01 | 0.936 |
| HBoV（n，%） | 13（2.1） | 7（2.8） | 0.50 | 0.919 |
| Bacteria（n，%） |  |  |  |  |
| *H. influenzae* | 83（23.1） | 47（28.1） | 1.55 | 0.213 |
| *Streptococcus pneumoniae* | 51（14.2） | 15（9.0） | 2.79 | 0.095 |
| *Staphylococcus aureus* | 23（6.4） | 13（7.8） | 0.35 | 0.554 |
| *Klebsiella pneumoniae* | 32（8.9） | 7（4.2） | 3.72 | 0.054 |

^a^HMPV: Human metapneumovirus.

^b^MP: *Mycoplasma pneumoniae*; HRV: Human rhinovirus; HAdV: Human adenovirus; HRSV: Human respiratory syncytial virus; HPIV: Human parainfluenza virus; FluB: Influenza B virus; HBoV: Human bocavirus; *H. influenza*:*Haemophilus influenza*.

**TABLE 3**. Comparison of treatment and length of hospital stay between single HMPV infection and co-infection groups in patients with severe pneumonia^a^

| Group | n | | Glucocorticoids  （n，%） | Human  immunoglobulin  （n，%） | Fiberoptic  bronchoscopy treatment  （n，%） | Antibiotics（n，%） | Length of hospital stay(IQR) |
| --- | --- | --- | --- | --- | --- | --- | --- |
| Single infection | 79 | 45（33.3） | | 8（32.0） | 5（11.4） | 78（31.8） | 7（5.5，8.5） |
| Co-infection | 167 | | 90（66.7） | 17（68.0） | 39（88.6） | 167（68.2） | 7（5，10） |
| χ^2b^ |  | | 0.204 | 0.000 | 10.950 | 2.123 | -0.610* |
| *P* |  | | 0.651 | 0.990 | 0.001 | 0.321 | 0.542 |

^a^HMPV: Human metapneumovirus. ^b^* represents the Z-value

**TABLE 4**. Seasonal and interannual distribution of mild and severe cases of HMPV^a^‑positive CAP, 2021-2024

| Year | Season^b^ | Mild pneumonia | Severe pneumonia |
| --- | --- | --- | --- |
| 2021 | Spring | 31(23.3) | 2(8.0) |
|  | Summer | 7(5.3) | 0(0.0) |
|  | Autumn | 6(4.5) | 0(0.0) |
|  | Winter | 89(66.9) | 23(92.0) |
| Total |  | 133 | 25 |
| 2022 | Spring | 21(15.4) | 9(14.5) |
|  | Summer | 1(0.7) | 0(0.0) |
|  | Autumn | 9(6.6) | 6(9.7) |
|  | Winter | 105(77.2) | 47(75.8) |
| Total |  | 136 | 62 |
| 2023 | Spring | 35(19.0) | 15(21.4) |
|  | Summer | 77(41.8) | 29(41.4) |
|  | Autumn | 45(24.5) | 18(25.7) |
|  | Winter | 27(14.7) | 8(11.4) |
| Total |  | 184 | 70 |
| 2024 | Spring | 64(35.8) | 29(32.6) |
|  | Summer | 4(2.2) | 0(0.0) |
|  | Autumn | 7(3.9) | 2(2.2) |
|  | Winter | 104(58.1) | 58(65.2) |
| Total |  | 179 | 89 |

^a^HMPV: Human metapneumovirus.

^b^Seasons are defined according to meteorological standards: spring (March-May), summer (June-August), autumn (September-November), and winter (December-February).
